# Dysfunction of small airways and prevalence, airway responsiveness and inflammation in asthma: much more than small particle size of pet animal allergens

**DOI:** 10.3109/03009734.2016.1173745

**Published:** 2016-07-10

**Authors:** Gennaro Liccardi, Antonello Salzillo, Amedeo Piccolo, Luigino Calzetta, Paola Rogliani

**Affiliations:** Department of Chest Diseases, Division of Pneumology and Allergology, High Speciality A. Cardarelli Hospital, Naples Italy Postgraduate School of Respiratory Medicine, Department of Systems Medicine, University of Rome Tor Vergata, Rome, Italy; Department of Chest Diseases, Division of Pneumology and Allergology, High Speciality A. Cardarelli Hospital, Naples Italy; Department of Systems Medicine, University of Rome Tor Vergata, Rome, Italy Postgraduate School of Respiratory Medicine, Department of Systems Medicine, University of Rome Tor Vergata, Rome, Italy

Dear Editor,

We read with interest the excellent article from Patelis et al. (1) showing that sensitization to small aeroallergens, produced by common pets, was associated with local airway and systemic inflammation, airway responsiveness, and higher prevalence of asthma. We believe that the topic of ‘animal allergy’ may be of great interest not only for clinicians but also for emotional implications in all pet-owner patients, especially in children and in atopic individuals who wish to own a pet. The love for animals in general, and for pets in particular, is increasing worldwide. Therefore, it is necessary to evaluate the role of exposure to pet allergens related with the risk factors inducing bronchial asthma, which may also involve small airways.

We were not surprised by the findings of Patelis et al. (1) considering the well-known capacity of smaller pet-allergen-carrying particles to reach peripheral airways and induce significant bronchoconstriction (2). In addition, the high rate of pet ownership in Northern Europe may further increase the direct exposure to pets. However, in our opinion, some important limitations to the conclusions of this research have not been included in the list of weaknesses already acknowledged by the authors.

First of all, it has been shown that dysfunction of the small airways must be considered in a distinct asthma phenotype (3) characterized by several clinical characteristics such as poor disease control, frequent exacerbations, and high degree of severity of bronchial hyper-responsiveness. It is important to underline that several non-allergenic and allergenic agents are able to reach peripheral airways, inducing inflammatory events similar to those determined by pet allergens. Outdoor air pollutants, such as PM2.5 and indoor cigarette smoke, represent the most common non-allergenic agents which determine a ‘synergistic interaction’ with allergic sensitization when these compounds are inhaled together with allergens. Moreover, they can also determine directly airway inflammation and asthma exacerbation in already diagnosed asthmatics (4,5).

Apart from pets, other allergenic sources can release allergens in ‘respirable’ (0.5–3 μm diameter) form such as dust mites (6), moulds (e.g. *Alternaria* and *Aspergillus* spp.) (7), and pollens (e.g. *Parietaria*, grasses, birch, *Olea europaea*, Artemisia) (8). In particular, pollen grains can induce so-called ‘thunderstorm asthma’ (9,10). In wet conditions, just before the onset of a thunderstorm, atmospheric pollens can release pauci-micronic particles containing allergens. The inhalation of these particles may induce severe asthma exacerbations in some highly sensitized individuals that, in turn, may even require emergency department visits or hospitalization.

In conclusion, we agree that about 20% of pet-allergen-carrying particles are characterized by aerodynamic properties of reaching small airways, and that these particles are likely to induce intensive inflammatory events. However, many other allergenic/non-allergenic agents are able to induce similar responses in association or not with pet allergens. In geographical areas with a lower rate of pet ownership (and a prevalent ‘indirect’ modality of exposure to pets), previously described agents are likely to be more decisive in inducing asthma with prevalent small airways involvement. Finally, dysfunction of small airways is now hypothesized also as a ‘remodelling phenotype’. In other words, individual subjects react to different environmental stimuli with a prevalent ‘peripheral’ response. Considering the relevant implications of pet–human relationships, especially in children, these considerations must be taken into account in order to avoid unjustified overestimation or generalization of the role of pet allergens in inducing a higher degree of asthma severity.
